# Extracellular Matrix Protein 1 Regulates Colorectal Cancer Cell Proliferative, Migratory, Invasive and Epithelial-Mesenchymal Transition Activities Through the PI3K/AKT/GSK3β/Snail Signaling Axis

**DOI:** 10.3389/fonc.2022.889159

**Published:** 2022-04-27

**Authors:** Sirui Long, Jie Wang, Fanbin Weng, Debing Xiang, Guiyin Sun

**Affiliations:** ^1^Departments of Oncology, Chongqing University Jiangjin Hospital, Chongqing, China; ^2^Departments of Oncology, Jiangjin Central Hospital of Chongqing, Chongqing, China

**Keywords:** Extracellular matrix protein 1, colorectal cancer, metastasis, epithelial-mesenchymal transition, PI3K/AKT/GSK3β/Snail signaling pathway

## Abstract

In prior reports, extracellular matrix protein 1 (ECM1) upregulation has been reported in colorectal cancer (CRC) patient tumor tissues, and has been suggested to be related to the metastatic progression of CRC, although the underlying mechanisms have yet to be clarified. In this study, we found that ECM1 was overexpressed in both CRC tissues and cell lines. Upregulation of ECM1 was correlated with tumor size, lymph node status and TNM stage in CRC patients. Knocking down ECM1 suppressed CRC cell growth, migration and invasion, in addition to reducing the expression of Vimentin and increasing E-cadherin expression. The overexpression of ECM1, in contrast, yielded the opposite phenotypic outcomes while also promoting the expression of p-AKT, p-GSK3β, and Snail, which were downregulated when ECM1 was knocked down. Treatment with LY294002 and 740 Y-P reversed the impact upregulation and downregulation of ECM1 on CRC cell metastasis and associated EMT induction. *In vivo* analyses confirmed that ECM1 overexpression was able to enhance EMT induction and CRC tumor progression. In conclusion, ECM1 influences CRC development and progression in an oncogenic manner, and regulates CRC metastasis and EMT processes *via* the PI3K/AKT/GSK3β/Snail signaling axis.

## Introduction

One of the most prevalent gastrointestinal malignancies in the world is colorectal cancer (CRC) (1). While 5-year survival rates for individuals with early-stage CRC are 80-90%, they decrease significantly to 40-60% among individuals with advanced-stage disease, and decline further to 5-10% for cases of metastatic CRC (2). Clarifying the molecular strategies governing the metastatic progression of CRC is thus crucial to identifying novel approaches to diagnosing this disease during its early stages and preventing its aggressive spread in patients.

The secretory glycoprotein, extracellular matrix protein 1 (ECM1), is associated with physiological processes including angiogenesis, the proliferation of epithelial cells, and skin differentiation, in addition to playing a role in tumor progression (3, 4). Recent studies have detected ECM1 overexpression in bladder cancer (5), glioblastoma (6), thyroid cancer (7), cholangiocarcinoma (8), and other epithelial malignancies (9), and the overexpression of ECM1 has also been shown to be related to the poor prognosis of cancer patients, such as pancreatic cancer (10), breast cancer (11), gastric cancer (12) and hepatocellular cancer patients (13). In prior reports, ECM1 mRNA and protein levels have been shown to be increased in CRC cancer tissue samples relative to normal tissues, with this upregulation being more pronounced in CRC patients exhibiting lymph node metastases (14, 15). The mechanisms underlying such upregulation, however, have yet to be clarified.

In the present study, we confirmed that ECM1 was overexpressed in CRC cancer tissues and cell lines. To further clarify the role of ECM1 in CRC, we performed *in vitro* and *in vivo* assays after the knockdown or overexpression of ECM1. We additionally explore the mechanisms whereby ECM1 regulates the growth, invasion, and migration of CRC cells and associated metastatic changes. Through this approach, we found that the regulation of the occurrence and progression of CRC by ECM1 was mediated through the PI3K/AKT/GSK3β/Snail signaling pathway.

## Methods

### Oncomine Data Collection

Oncomine (www.oncomine.org) was used to compare ECM1 expression levels between CRC tissues and normal tissues.

### Patient Recruitment and Sample Collection

Tissue samples were obtained from CRC patients undergoing radical surgical treatment at Jiangjin Central Hospital of Chongqing between January 2020 and June 2021, with 65 CRC tumor tissue samples and 60 paired paracancerous tissue samples being collected, formalin-fixed, and paraffin-embedded. In addition, 10 fresh pairs of CRC patient tissues were collected between August 2021 and December 2021 and were stored at -80°C. Samples were obtained from patients with histologically confirmed diagnoses that had not undergone preoperative surgical, immunotherapeutic, or chemotherapeutic treatment. The Institutional Ethics Committee of Jiangjin Central Hospital approved present study(KY20200010), with all contributors having presented the letter of informed consent to participate.

### Immunohistochemistry

Formalin-fixed paraffin-embedded tissues were sliced to prepare 3 μm thick sections. Antigen retrieval was conducted using citrate buffer (pH = 6; ZSGB-BIO, ZLI-9064) based on the protocols included in an IHC staining kit (ZSGB-BIO, PV-9000). Slides were probed overnight with primary anti-ECM1(1:250; Abcam) at 4°C, followed by incubation with an appropriate secondary antibody at room temperature for 30 min. In negative control sections, PBS was employed in lieu of primary antibody. As per a previous study (12), IHC scoring was conducted by assessing the percentage of stained cells (1 = < 25%, 2 = 25-50%, 3 = 51-75%, and 4 = >75%) and multiplying this score by the staining intensity (0, 1, 2, or 3) for a final possible score of 0-12. Scores < 4 were considered to indicate low levels of ECM1 expression, while all other scores were indicative of high expression. Two experienced pathologists blinded to patient characteristics independently scored all samples.

### Cell Culture

NCM460, HCT116, HCT15, HT29, SW480, 293T, and SW620 were purchased from BeNa Culture Collection (Beijing, China). NCM460 and 293T cells were grown in HG media (Gibco), while SW620, HCT116, HT29, SW480, and HCT15 cells were grown in RPMI-1640 (Gibco) including penicillin/streptomycin (Gibco) and 10% FBS (Gibco). All cells were cultured in a humidified 5% CO_2_ incubator at 37°C.

### Transfection

Cells were added to 6-well plates (2× 10^5^/well) and grown to 40-60% confluence, at which time Lipofectamine 2000 (Invitrogen) was used to transfect cells with appropriate siRNAs (Genepharma), followed by incubation for 6 h in serum-free media, after which media was exchanged for media containing 10% FBS. Following an additional 24 h incubation, qPCR was performed to assess ECM1 knockdown efficiency, with Western blotting and cell functional assays being performed at 48 h post-transfection.

siRNA NC sense: 5’-UUCUCCGAACGUGUCACGUTT-3’, antisense: 5’-ACGUGACACGUUCGGAGAATT-3’; siRNA-1 sense: 5’-GAAGCAAUGAGCCGAUUCUTT-3’, antisense: 5’-AGAAUCGGCUCAUUGCUUCTT-3’; siRNA-2 sense: 5’-GUGGAAACCAAAGAGUUCUTT-3’, antisense: 5’-AGAACUCUUUGGUUUCCACTT-3’; siRNA-3 sense: 5’-GAGGAGAAAUUAACCUUCATT-3’, antisense: 5’-UGAAGGUUAAUUUCUCCUCTT-3’.

### Lentiviral Transduction

HEK293T cells were co-transfected with the pLVX-CMV-IRES-EGFP-puro empty vector or pLVX-CMV-IRES-EGFP-puro-ECM1 vector along with lentiviral packaging plasmids (psPAX2 and pMD2.G). After 72 h, supernatants were collected, centrifuged for 20 min at 3000 rpm, filtered, centrifuged at 4°C for 20 min at 5000 rmp and concentrated. The resultant lentiviral concentrates were then used to infect cells, generating cell lines expressing ECM1 or the empty vector control.

### qRT-PCR

The Tsingzol RNA Extraction Reagent (Tsingke, TSP401) was utilized to extract RNA from cells and tissue samples, after which cDNA was produced with a ReverTra Ace^®^ qPCR RT Kit (TOYOBO, FSQ-101). Subsequent qPCR reactions were performed employing SYBR^®^ Green Realtime PCR Master Mix (TOYOBO, QPK-201) with an Applied Biosystems 7300Plus Real-Time PCR Instrument (Applied Biosystems, MA, USA). The 2^-ΔΔCt^ approach was utilized for the assessment of relative gene expression, with analyses being conducted in triplicate. Utilized primers are as follows: β-actin-F: 5’-ACCCCGTGCTGCACCGAG-3’, β-actin-R: 5’-TCCCGGCCAGCCAGGTCCA-3’; ECM1-F: 5’-CCTGGGCTGATCCACAACAT-3’; ECM1-R:5’-TTGGCGTTCTCAGTGTCTCC-3’.

### Western Blotting

RIPA Buffer (Beyotime Biotechnology, China) containing 1% PMSF (Thermo, USA) was used to extract protein from cell and tissue samples, after which a BCA Kit (Beyotime) was employed to measure protein concentrations. Equal protein amounts from individual samples (cells: 30 ug; tissues: 60 ug) were subsequently separated *via* 10% SDS-PAGE and transferred to PVDF membranes (Millipore) that were then blocked for 2 h with 5% non-fat milk at room temperature, followed by overnight incubation with primary antibodies diluted in TBST containing 5% non-fat milk at 4°C. Blots were then incubated at room temperature with HRP-conjugated anti-rabbit or anti-mouse IgG (ZSBIO, China), after which a chemiluminescent imaging system was used for protein band detection. Primary antibodies included antibodies specific for the following, all of which were diluted at 1:1000 before use: ECM1 (Abcam, UK), E-cadherin (CST, USA), Vimentin (CST, USA), Snail (CST, USA), AKT (CST, USA), p-AKT (Ser473) (CST, USA), GSK3β (Novus, USA), p-GSK3β (Ser9) (Novus, USA), GAPDH (CST, USA). Image J was used for quantitative analyses.

### Cell Proliferation Assays

CCK-8 and colony formation assays were used to appraise cellular proliferation. Briefly, cells were added to 96-well plates (5000 cells/well in 100 uL) and incubated until adherent. CCK-8 reagent (10 uL; CK04, Tongren Company, Japan) was then added to each well after 0, 24, 48, or 72 h, followed by an additional 2 h incubation at 37°C. Absorbance at 450 nm was subsequently analyzed with a microplate reader (FLUOstar Omega, BMG LABTECH).

### Colony Formation Assay

Cells were added to 6-well plates (1000/well) and cultured until colony formation was evident, at which time 4% paraformaldehyde was used to fix colonies which were then stained for 1 min with 0.2% crystal violet at ambient temperature. Colonies were then counted using ImageJ.

### Cell Cycle and Apoptosis

To assess cell cycle progression, cells were added to 6-well plates (3× 10^5^/well) and cultivated for 24 h, at which time they were harvested using trypsin, rinsed with PBS, and fixed for 24 h with 70% ethanol at 4°C. Cells were subsequently stained with an AnnexinV/PI double staining kit (BD, USA) based on provided directions, followed by analysis *via* flow cytometry (BD, USA). Apoptotic cell death was assessed by plating cells under the same conditions, and then harvesting and rinsing these cells three times using PBS prior to resuspension in 100 uL of binding buffer. Cells were next stained with 5 uL of Annexin V-FITC and 10 uL of PI for 15 min on ice, after which 400 uL of binding buffer was added. Cells were analyzed within 1 h *via* flow cytometry (BD, USA).

### Transwell Assays

Transwell polycarbonate inserts (8 μm pore size) that were or were not coated with Matrigel (plain RPMI-1640:Matrigel = 9:1; BD) were used with 24-well plates to assess cellular migration and invasion. Cells (2x10^5^ in 200 uL) were added to the upper chamber of each well, while 600 uL of RPMI-1640 contaning 20%FBS was added to the lower chamber. Following culture for 24 h, cells were fixed with 4% paraformaldehyde for 30 min, stained for 1 min with crystal violet, and non-migratory/invasive cells were eliminated with a cotton swab. Those cells bound to the lower chamber surface were then imaged *via* microscopy, with three random fields of view per sample being assessed.

### Wound-Healing Assays

Cells were added to 6-well plates (1×10^6^/well) and were grown until 90% confluent, at which time a 200 uL pipette tip was used to generate a scratch wound in the monolayer surface. Cells were then rinsed with PBS and cultured in RPMI-1640 containing 1%FBS for 24 h, with the rate of wound healing (%) being determined by measuring the area between the two sides of the wound using ImageJ. Wound healing rate = (area at 0 h - area at 24 h)/area at 0 h.

### Xenograft Mouse Model

Male BALB/c nude mice (4 weeks old, Laboratory Animal Center of Shanghai, Shanghai, China) were housed in a temperature-controlled facility with a natural day/night cycle and free access to water and food. Laboratory Animal Welfare and Ethics Committee of Chongqing University approved all animal studies, which were performed in a manner consistent with the Guide for the Care and Use of Laboratory Animals. A xenograft tumor model was established by subcutaneously injecting mice in the right flank with HCT116-NC or HCT116-ECM1 cells (2×10^6^ cells/50ul). On day 25 post-inoculation, mice were anesthetized and humanely euthanized, at which time tumors were weighed and collected for downstream analyses.

### Statistics Analysis

Statistical analyses were performed using SPSS 26.0, GraphPad Prism 9.0, and ImageJ. Differences were compared between groups using two-tailed t-tests, while the relationships between ECM1 expression and clinicopathological characteristics were evaluated *via* Pearson χ2 analyses. *In vitro* studies were repeated in triplicate and reported as means ± standard deviation (x ± s). *P<0.05, **P<0.01, ***P<0.001, and ****P<0.0001.

## Results

### ECM1 Is Upregulated in CRC Tumor Tissues and Cell Lines

**Figure 1:**
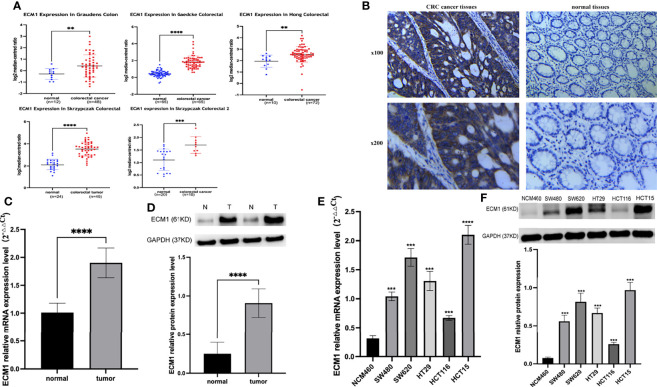
Analysis of ECM1 expression in CRC tumors and cell lines. **(A)** ECM1 levels in CRC patient tumors and adjacent normal tissue samples were analyzed using the Oncomine database. **(B)** Representative IHC staining results for CRC patient tumor and paracancerous tissues. **(C)** ECM1 mRNA expression in CRC tumors and paracancerous tissues. **(D)** ECM1 protein levels in CRC tumors and paracancerous tissues. **(E)** ECM1 mRNA levels in CRC and colonic epithelial cell lines. **(F)** ECM1 protein levels in CRC (SW480, SW620, HT29, HCT116, HCT15) and colonic epithelial (NCM460) cell lines. Data are means ± SD (**P < 0.01, ***P < 0.001, ****P < 0.0001).

An initial analysis of the Oncomine database revealed ECM1 to be highly upregulated in CRC patient tumor tissues as compared to normal tissue samples (**Figure 1A**). Subsequent IHC, qPCR, and Western blotting analyses similarly confirmed the upregulation of ECM1 in CRC tumors relative to normal paracanceorus tissues (**Table 1** and **Figures 1B–D**). IHC scores were then employed to evaluate the association between ECM1 expression and CRC patient clinicopathological features, revealing the expression of ECM1 to be significantly associated with CRC patient TNM staging (*P*<0.001), lymph node metastasis (*P*<0.001), and tumor size (*P*<0.05) (**Tables 2**, **3**). Consistent with the above results, higher levels of ECM1 expression were observed in CRC cell lines (SW480, HT29, HCT15, HCT116, SW620) relative to those in normal colonic epithelial cells (NCM460) at both the protein and mRNA levels (**Figures 1E, F**).

**Table 1:** ECM1 expression levels in CRC tumors and paracancerous tissue samples.

Group	Number	ECM1		*x^2^*	*P*
		High expression	Low expression		
Tumor	65	39	26	33.85	<0.0001
Normal	60	6	54

**Table 2:** Associations between ECM1 expression and CRC patient clinicopathological features.

Clinicopathological features	High (n = 39)		Low (n = 26)		*P*
	N %		N %		
Gender					
male	24	61.54	15	57.69	0.756
female	15	38.46	11	42.31
Age					
< 60y	8	20.51	5	19.23	0.899
≥ 60y	31	79.49	21	80.77
Tumor location					
right hemicolon	10	25.64	9	34.62	0.718
left hemicolon	13	33.33	7	35.00
rectum	16	41.03	10	30.38
Differentiation					
Moderate and well	27	69.23	18	69.23	1.000
Poor	12	30.77	8	30.77
Tumor size					
< 5cm	17	43.59	18	69.23	0.042
≥ 5cm	22	56.41	8	30.77
Lymph node metastasis					
–	13	33.33	19	73.08	0.002
+	26	66.67	7	26.92
Distant metastasis					
–	31	79.49	24	92.31	0.160
+	8	20.51	2	7.69
TNM					
I-II	12	30.77	18	69.23	0.002
III-IV	27	69.23	8	30.77

**Table 3:** Patients’ clinical information and ECM1 expression level.

Number	Age	Gender	Tumor location	Differentiation	Tumor size	T	Lymph node number	N	Distant metastasis	M	TNM	TNM stage	Cancer tissue (ECM1 expression level) (n=65)	Normal tissue (ECM1 expression level) (n=60)
1	76	female	right hemicolon	poor	4.5cm	T3	0	N0	–	M0	T3N0M0	II	low	low
2	78	female	rectum	moderate and well	2cm	T2	0	N0	–	M0	T2N0M0	I	low	low
3	64	female	rectum	moderate and well	4cm	T2	0	N0	+	M1	T4N0M1	IV	high	low
4	69	female	rectum	moderate and well	2.5cm	T2	0	N0	–	M0	T2N0M0	I	low	low
5	74	male	rectum	poor	5.5cm	T2	3	N1	–	M0	T2N1M0	II	low	low
6	66	male	right hemicolon	moderate and well	7cm	T3	0	N0	–	M0	T3N0M0	II	low	–
7	77	male	rectum	moderate and well	4.5cm	T2	0	N0	–	M0	T2N0M0	I	low	low
8	51	male	rectum	moderate and well	4cm	T4	1	N1	–	M0	T4N1M0	III	high	high
9	70	male	rectum	moderate and well	5.5cm	T4	6	N2	–	M0	T4N2M0	III	high	low
10	77	female	right hemicolon	moderate and well	5cm	T3	3	N1	–	M0	T3N1M0	III	low	low
11	66	female	left hemicolon	moderate and well	3cm	T3	3	N1	–	M0	T3N1M0	III	high	low
12	70	male	rectum	moderate and well	7cm	T4	1	N1	–	M0	T4N1M0	III	high	low
13	63	male	rectum	moderate and well	5cm	T4	10	N2	–	M0	T4N2M0	III	high	high
14	69	male	left hemicolon	moderate and well	4cm	T4	3	N1	–	M0	T4N1M0	III	low	low
15	47	female	rectum	moderate and well	4.5cm	T4	5	N2	–	M0	T4N2M0	III	high	low
16	75	male	right hemicolon	moderate and well	4cm	T3	0	N0	–	M0	T3N0M0	II	low	low
17	56	male	left hemicolon	moderate and well	3.5cm	T3	0	N0	–	M0	T3N0M0	II	low	low
18	71	male	rectum	moderate and well	5cm	T4	0	N0	–	M0	T4N0M0	II	low	low
19	65	male	rectum	moderate and well	4.5cm	T4	0	N0	–	M0	T4N0M0	II	high	–
20	75	male	rectum	moderate and well	4cm	T4	3	N1	–	M0	T4N1M0	III	high	low
21	61	male	right hemicolon	poor	3.5cm	T3	0	N0	–	M0	T3N0M0	II	low	low
22	76	female	rectum	moderate and well	2.5cm	T1	0	N0	–	M0	T1N0M0	I	low	low
23	74	male	left hemicolon	moderate and well	4.5cm	T3	0	N0	–	M0	T3N0M0	II	high	high
24	82	male	rectum	moderate and well	2.5cm	T2	0	N0	–	M0	T2N0M0	I	low	low
25	68	male	rectum	moderate and well	5.5cm	T4	0	N0	–	M0	T4N0M0	II	low	low
26	59	female	rectum	moderate and well	4.5cm	T4	0	N0	–	M0	T4N0M0	II	high	low
27	80	male	rectum	moderate and well	4cm	T4	0	N0	+	M1	T4N0M1	IV	low	–
28	80	male	rectum	poor	3.5cm	T4	7	N2	–	M0	T4N2M0	III	high	low
29	66	male	left hemicolon	poor	4cm	T3	1	N1	–	M0	T3N1M0	III	high	high
30	54	female	right hemicolon	moderate and well	4cm	T3	0	N0	–	M0	T3N0M0	II	high	low
31	69	male	right hemicolon	moderate and well	3cm	T3	3	N1	+	M1	T3N1M1	IV	high	high
32	71	male	left hemicolon	poor	5cm	T3	3	N1	+	M1	T3N1M1	IV	high	low
33	72	female	rectum	moderate and well	2.5cm	T2	2	N1	–	M0	T2N1M0	II	high	low
34	86	female	left hemicolon	poor	3.5cm	T3	5	N2	–	M0	T3N2M0	III	high	low
35	64	male	rectum	moderate and well	5cm	T4	3	N1	–	M0	T4N1M0	III	high	low
36	49	female	right hemicolon	moderate and well	6cm	T3	0	N0	–	M0	T3N0M0	II	high	low
37	59	male	rectum	moderate and well	10cm	T4	0	N0	–	M0	T4N0M0	II	high	low
38	80	female	right hemicolon	poor	5cm	T3	2	N1	–	M0	T3N1M0	III	high	low
39	59	male	left hemicolon	poor	5cm	T3	0	N0	–	M0	T3N0M0	II	low	low
40	75	male	right hemicolon	poor	8cm	T3	0	N0	–	M0	T3N0M0	II	high	low
41	54	female	left hemicolon	poor	5.5cm	T3	5	N2	–	M0	T3N2M0	III	high	low
42	70	male	left hemicolon	poor	5cm	T4	0	N0	+	M1	T4N0M1	IV	high	low
43	69	male	rectum	moderate and well	5cm	T4	1	N1	–	M0	T4N1M0	III	high	low
44	66	male	rectum	moderate and well	3cm	T3	0	N0	+	M1	T3N0M1	IV	low	low
45	78	male	right hemicolon	moderate and well	5cm	T3	0	N0	–	M0	T3N0M0	II	high	low
46	80	male	right hemicolon	poor	10cm	T3	0	N0	–	M0	T3N0M0	II	low	–
47	72	male	left hemicolon	moderate and well	7cm	T3	5	N2	–	M0	T3N2M0	III	high	low
48	64	female	left hemicolon	moderate and well	3cm	T3	0	N0	–	M0	T3N0M0	II	low	low
49	68	male	left hemicolon	poor	5cm	T4	9	N2	+	M1	T4N2M1	IV	high	low
50	70	male	rectum	moderate and well	4cm	T4	9	N2	–	M0	T4N2M0	III	high	low
51	57	female	right hemicolon	moderate and well	4cm	T2	0	N0	–	M0	T2N0M0	I	low	low
52	52	female	left hemicolon	moderate and well	3cm	T3	3	N1	–	M0	T3N1M0	III	high	low
53	44	female	right hemicolon	poor	4cm	T3	4	N2	–	M0	T3N2M0	III	low	low
54	81	female	left hemicolon	moderate and well	5cm	T3	0	N0	–	M0	T3N0M0	II	high	low
55	81	female	right hemicolon	moderate and well	8cm	T3	0	N0	–	M0	T3N0M0	II	high	high
56	72	male	right hemicolon	poor	5cm	T3	2	N1	–	M0	T3N1M0	III	low	low
57	65	female	left hemicolon	moderate and well	4.5cm	T3	0	N0	–	M0	T3N0M0	II	low	low
58	49	female	left hemicolon	poor	4cm	T3	10	N2	–	M0	T3N2M0	III	low	–
59	69	male	right hemicolon	moderate and well	6cm	T4	7	N2	+	M1	T4N2M1	IV	high	low
60	64	male	rectum	poor	4.5cm	T4	3	N1	–	M0	T4N1M0	III	high	low
61	60	female	right hemicolon	poor	5cm	T3	11	N2	–	M0	T3N2M0	III	high	low
62	83	male	left hemicolon	moderate and well	7cm	T3	31	N2	+	M1	T3N2M1	IV	high	low
63	73	female	left hemicolon	moderate and well	4cm	T3	7	N2	–	M0	T3N2M0	III	low	low
64	84	female	left hemicolon	moderate and well	5cm	T3	0	N0	–	M0	T3N0M0	II	high	low
65	84	male	right hemicolon	poor	5cm	T3	19	N2	+	M1	T3N2M1	IV	high	low

### ECM1 Regulates the *In Vitro* Proliferation of CRC Cells

**Figure 2:**
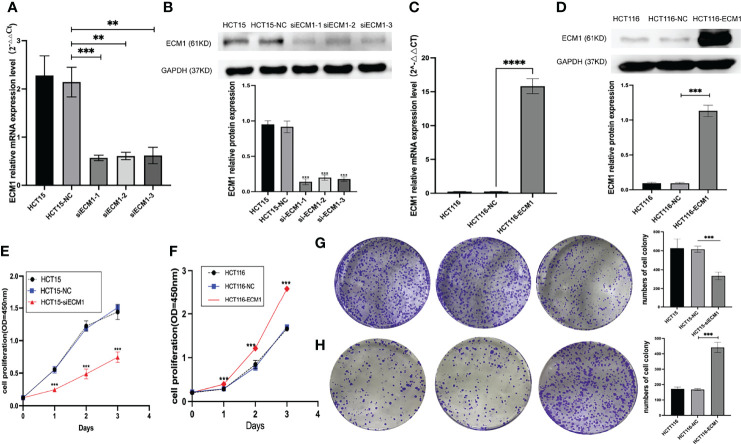
ECM1 regulates proliferative activity in CRC cells. **(A, B)**. Western blotting and qPCR were performed to assess ECM1 knockdown. **(C, D)**. Western blotting and qPCR were employed to assess ECM1 expression in CRC cells following its overexpression. CCK-8 and colony formation assays were used to assess cellular proliferation, revealing that **(E, G)** ECM1 knockdown suppressed HCT15 cell proliferation relative to control cells, while **(F, H)** ECM1 overexpression enhanced HCT116 cell proliferation. Data are means ± SD (**P < 0.01, ***P < 0.001, ****P < 0.0001).

As the highest and lowest levels of ECM1 were respectively observed in HCT15 and HCT116 cells, ECM1 was next knocked down and overexpressed in these respective cell lines. Both qPCR and Western blotting were used to confirm effective knockdown using three different ECM1-specific siRNA constructs (**Figures 2A, B**), with siRNA-1 being used for subsequent studies. Similarly, qPCR and Western blotting were used to confirm the efficiency of ECM1 overexpression, (**Figures 2C, D**). Colony formation and CCK-8 assays were then conducted to assess cellular proliferation, revealing that ECM1 knockdown significantly impaired the proliferative ability of these CRC cells, whereas its overexpression enhanced such proliferation relative to that observed for control cells (**Figures 2E** **–H**). Together, the achieved findings suggest that ECM1 is able to promote the *in vitro* proliferation of CRC cells.

### ECM1 Influences *In Vitro* CRC Cell Apoptosis and Cell Cycle Progression

**Figure 3:**
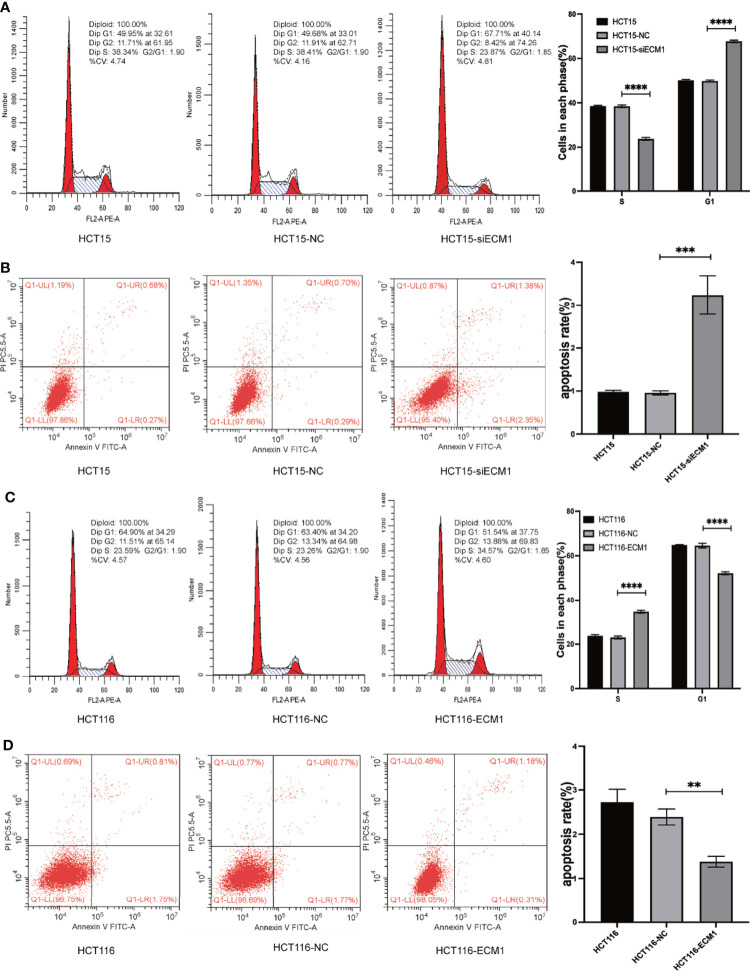
ECM1 regulates the progression of the cell cycle and apoptotic death in CRC cells. Following ECM1 knockdown or overexpression, apoptosis and cell cycle progression were analyzed *via* flow cytometry. **(A, B)** Changes in the progression of the cell cycle and apoptotic cell death following the knockdown of ECM1. **(C, D)** Changes in the progression of the cell cycle and apoptotic cell death following ECM1 overexpression. Data are means ± SD (**P < 0.01, ***P < 0.001, ****P < 0.0001).

The ability of ECM1 to regulate cell cycle and apoptotic death in CRC cells was next evaluated *via* flow cytometry. In these assays, ECM1 knockdown enhanced the frequency of cells in the G1 phase while reducing the frequency of cells in the S phase (**Figure 3A**), with a increase in the rate of apoptotic death among HCT15-siECM1 cells as compared to HCT15 and HCT15-NC cells (**Figure 3B**). These results thus confirmed that ECM1 knockdown was readily able to suppress *in vitro* CRC cell growth. In contrast, ECM1 upregulation enhanced the proliferative activity of these cells while reducing the frequency of apoptotic death (**Figures 3C, D**). Together, these data thus confirmed that ECM1 can serve as a regulator of cell cycle progression and apoptotic death in this cancer type.

### ECM1 Regulates the *In Vitro* Invasivity and Migratory Activity of CRC Cells

**Figure 4:**
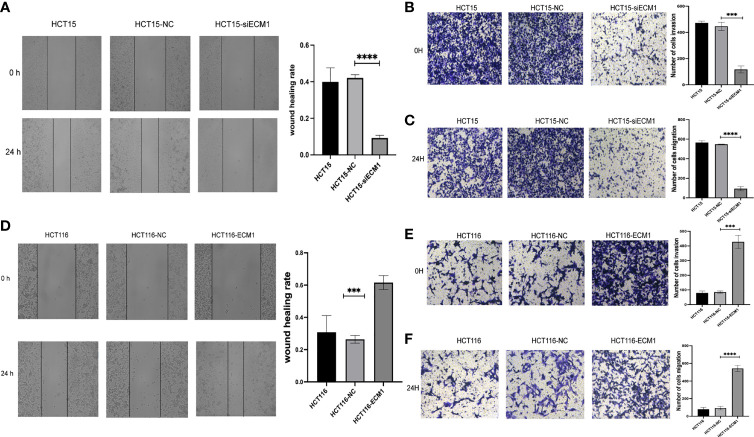
ECM1 regulates CRC cell invasion and migration. CRC cell migratory and invasive activity were scrutinized through Transwell and wound healing assessments following ECM1 knockdown **(A–C)** or upregulation **(D–F)**. Data are means ± SD (***P < 0.001, ****P < 0.0001).

Next, the role of ECM1 as a regulator of migratory and invasive activity was assessed through Transwell and wound healing assays. These analyses revealed HCT15 cell invasivity and migratory activity to be significantly suppressed upon ECM1 knockdown relative to control HCT15 and HCT15-NC cells (**Figures 4A** **–C**), whereas the opposite phenotype was observed upon ECM1 overexpression (**Figures 4D** **–F**). These results thus indicated that ECM1 can promote CRC cell invasivity and migration in addition to enhancing their cell growth.

### ECM1 Is Associated With EMT Induction

**Figure 5:**
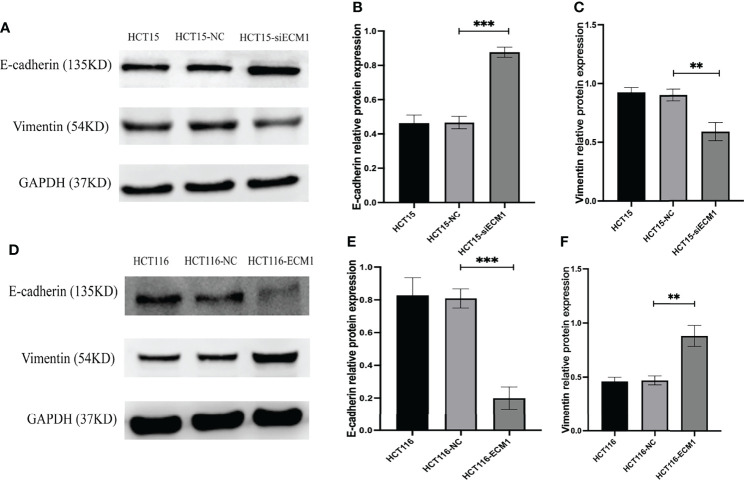
ECM1 regulates CRC cell EMT induction. EMT-related protein expression was analyzed following the knockdown or upregulation of ECM1. **(A–C)** Vimentin and E-cadherin protein levels were assessed in HCT15, HCT15-NC, and HCT15-siECM1 cells. **(D–F)** Vimentin and E-cadherin protein levels were investigated in HCT116, HCT116-NC, and HCT116-ECM1 cells. Data are means± SD (**P < 0.01, ***P < 0.001).

The EMT process plays a key role in promoting metastatic tumor progression. To analyze the link between ECM1 and this process, we conducted Western blotting assays examining EMT marker protein expression in CRC cells, revealing ECM1 knockdown to enhance E-cadherin expression while suppressing Vimentin expression (**Figures 5A** **–C**), whereas the opposite changes were observed upon ECM1 overexpression (**Figures 5D** **–F**). As such, ECM1 may regulate CRC progression at least in part *via* regulating EMT induction.

### ECM1 Modulates the PI3K/AKT/GSK3β/Snail Signaling Axis in CRC Cells

**Figure 6:**
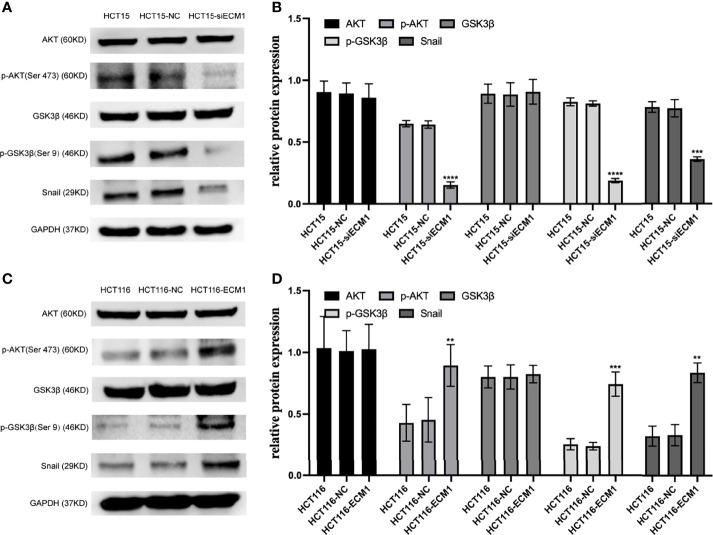
ECM1 regulates signaling *via* the PI3K/AKT/GSK3β/Snail axis within CRC cells. Western blotting was used to assess AKT, p-AKT, GSK, pGSK3β, and Snail levels in CRC cells following **(A, B)** ECM1 knockdown or **(C, D)** ECM1 overexpression. Data are means ± SD (**P < 0.01, ***P< 0.001, ****P < 0.0001).

In prior reports, signaling through the PI3K/AKT/GSK3β/Snail axis has been reported to be integral to EMT induction and consequent tumor progression. To further interrogate the mechanistic link between ECM1 and EMT induction in CRC cells, we thus analyzed the levels of AKT, GSK3β, p-AKT, p-GSK3β, and Snail following ECM1 knockdown or upregulation. Knocking down ECM1 reduced the phosphorylation of AKT and GSK3β as well as Snail expression without impacting total AKT or GSK3β expression (**Figures 6A, B**). In contrast, significant increases in p-AKT, p-GSK3β, and Snail levels were observed upon ECM1 upregulation (**Figures 6C, D**).

### PI3K/AKT Agonist and Inhibitor Treatment Can Reverse the Effects of Altered ECM1 Expression on CRC Cells

**Figure 7:**
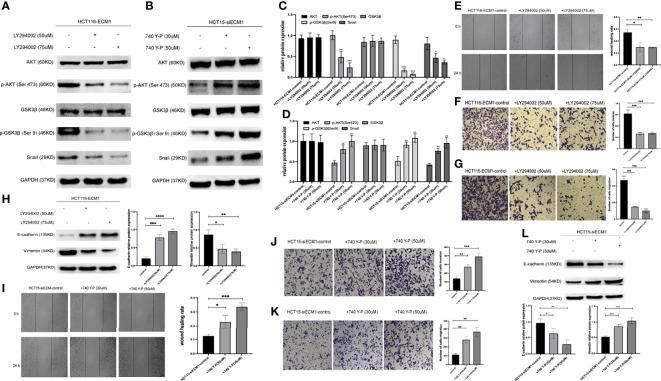
PI3K/AKT agonist and inhibitor treatment can reverse the effects of altered ECM1 expression on CRC cells. HCT116-ECM1 were treated for 24 h with LY294002 (50 or 75 uM), or HCT15-siECM1 were treated 24 h with 740 Y-P (30 or 50 uM), after which invasive and migratory activity were assessed through Transwell and wound healing assays **(E–G, I–K)**, while Western blotting was used to assess AKT, p-AKT, GSK, pGSK, Snail, E-cadherin, and Vimentin expression **(A–D, H,L)**. Data are means ± SD (*P < 0.05, **P < 0.01, ***P < 0.001, ****P <0.0001).

Next, CRC cells were treated with the PI3K/AKT inhibitor LY294002 to determine whether the ability of ECM1 to mediate EMT induction is tied to PI3K/AKT/GSK3β/Snail signaling pathway activation. Western blotting was used to detect changes in signaling-related protein levels (**Figures 7A, C**). These analyses revealed that the LY294002 treatment of HCT116-ECM1 cells suppressed their invasive and migratory activity in Transwell and wound healing assays (**Figures 7E** **–G**), and PI3K/AKT inhibitor treatment further suppressed EMT induction in these cells (**Figure 7H**). In contrast, the opposite changes were observed when HCT15-siECM1 cells were treated with PI3K/AKT agonist 740 Y-P (**Figures 7B, D** **, I–L**). Together, these results thus demonstrate the ability of ECM1 to induce CRC metastatic progression *via* the PI3K/AKT/GSK3β/Snail pathway.

### ECM1 Influences the *in vivo* Growth and EMT Induction of CRC Tumor Xenografts

**Figure 8:**
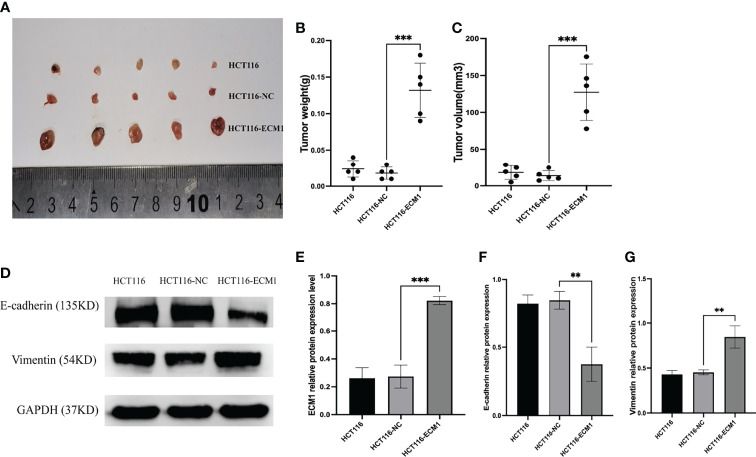
ECM1 promotes *in vivo* CRC tumor growth. **(A–C)** ECM1 enhanced *in vivo* tumor growth, as determined through measurements of tumor weight and volume at 25 days post-implantation. **(D–G)** ECM1, Vimentin, and E-cadherin expression levels in xenograft tumors were assessed *via* Western blotting. Data are means ± SD (**P < 0.01, ***P < 0.001)

Lastly, to examine the mechanisms whereby ECM1 influences *in vivo* CRC tumor growth, we generated a xenograft model system using nude mice implanted with HCT116, HCT116-NC, or HCT116-ECM1 cells (n=5 mice/group). ECM1 upregulation was associated with a significant increase in both tumor volume and weight (**Figures 8A** **–C**), while Western blotting revealed increased Vimentin expression and reduced E-cadherin expression in ECM1-overexpressing tumors (**Figures 8D–G**). These data thus confirmed that the upregulation of ECM1 can promote CRC progression and EMT induction *in vivo*.

## Discusion

At present, tumor metastasis and recurrence are the leading drivers of mortality among CRC patients (16, 17). However, the molecular mechanisms that govern the invasivity and migratory activity of CRC cells have yet to be fully clarified, underscoring the importance of analyzing novel regulators that can influence malignant tumor development and progression in an effort to highlight new approaches to the early diagnosis and treatment of this deadly tumor type.

The 85 kDa glycoprotein ECM1 was initially identified as a factor secreted by the MN7 murine osteogenic stromal cell line (18). More recent studies, however, have emphasized the functional importance of ECM1 as a regulator of tumor development, progression, and recurrence. Consistently, we herein analyzed the Oncomine database and found ECM1 to be upregulated in CRC patient tissues relative to normal tissues, with this finding subsequently being confirmed in an independent set of patient samples through qPCR, Western blotting, and IHC staining. Moreover, we found ECM1 to be closely correlated with CRC patient tumor size, TNM staging and lymph node metastasis. In line with these results, we observed ECM1 upregulation in CRC tumor cells relative to colonic epithelial cells at the mRNA and protein levels, and found that the overexpression of ECM1 was associated with enhanced CRC cell proliferative activity in CCK-8 and colony formation assays, whereas knocking down ECM1 yielded the opposite phenotypes. Subsequent flow cytometry analyses indicated that ECM1 knockdown increased the frequency of cells in the G1 cell cycle phase while reducing the frequency of cells in the S phase, with a corresponding increase in the frequency of apoptotic cell death. In contrast, overexpressing ECM1 suppressed apoptotic cell death while driving enhanced cell growth. Finally, we established a subcutaneous CRC tumor xenograft model system which demonstrated the ability of ECM1 overexpression to promote enhanced *in vivo* tumor growth, suggesting that ECM1 functions in an oncogenic manner in CRC.

The EMT process is central to in the metastatic progression of tumors of epithelial origin (19). Through EMT induction, epithelial cells undergo cytoskeletal reorganization, engage in extracellular matrix (ECM) remodeling, and become more mobile and invasive, detaching from surrounding cells and the ECM (20). These changes coincide with a reduction in the expression of epithelial marker proteins and the upregulation of mesenchymal marker proteins including Vimentin and N-cadherin, with this process being regulated by key transcription factors including members of the TWIST, SNAIL, and ZEB families (21, 22). Prior studies have shown ECM1 to play a role in this EMT process in several epithelial malignancies, thereby promoting metastatic progression. In gastric cancer, for example, ECM1 can promote ITGB4/GSK3β/SOX2/HIF-1α signaling and associated EMT induction (23), while in breast cancer it has been posited to stabilize β-catenin *via* MUC1, ultimately contributing to the EMT induction, the reinforcement of cancer stem cell phenotypes and associated tumor metastasis (24). As such, we hypothesize that ECM1 may similarly enhance CRC cell invasivity and migratory activity through EMT-associated mechanisms. To test this hypothesis, we conducted Transwell and wound healing assays which revealed ECM1 overexpression to promote enhanced migratory and invasive activity, whereas the opposite outcome was observed upon ECM1 downregulation. When EMT-related proteins were further evaluated *via* Western blotting, ECM1 overexpression was found to enhance and reduce the expression of Vimentin and E-cadherin, respectively. Through the establishment of ECM1-overexpressing tumor xenograft mouse models, we further confirmed that ECM1 overexpression in HCT116 cells *in vivo* was associated with consistent increases in Vimentin expression together with E-cadherin downregulation, suggesting that ECM1 can regulate the EMT process and thereby promote CRC cell metastatic progression *in vitro* and *in vivo*.

We additionally assessed the mechanisms whereby ECM1 can regulate metastatic activity in CRC cells. PI3K/AKT signaling axis is integral to tumor progression owing to its ability to enhance mobility and reduce intercellular adhesion (25). The EMT process and associated loss of epithelial cell phenotypes can be regulated in response to particular extracellular signaling molecules, with cell surface receptors and transcription factors ultimately transducing these signals to alter cellular characteristics (26, 27). The PI3K/AKT/GSK3β/Snail signaling has previously been shown to be critical to tumor metastasis *via* the modulation of EMT induction in multiple cancers (28–30). As such, we herein assessed the ability of ECM1 to affect the migratory and invasive activity of CRC cells *via* this signaling pathway, revealing that ECM knockdown was associated with decreases in p-AKT, p-GSK3β, and Snail expression in CRC cells, whereas ECM overexpression yielded the opposite outcome. When ECM1-overexpressing cells were treated with the PI3K/AKT inhibitor LY294002, this was additionally sufficient to suppress their migratory and invasive activity with concomitant vimentin downregulation and E-cadherin upregulation. In contrast, the opposite changes were observed when HCT15-siECM1 cells were treated with PI3K/AKT agonist 740 Y-P.

## Conclusion

In summary, we herein found ECM1 to be overexpressed in CRC patient tumors and CRC cell lines, with the expression of this oncogenic factor being related to patient TNM staging, lymph node metastasis, and tumor size. ECM1 overexpression was able to enhance CRC cell growth, migration, and invasion, whereas the opposite changes were observed upon ECM1 knockdown. Mechanistically, ECM1 was found to promote CRC metastatic progression *via* modulating EMT induction, potentially through regulating the PI3K/AKT/GSK3β/Snail signaling axis. Together, these results suggest ECM1 to be a promising candidate biomarker in CRC that has the potential to guide patient management, in addition to representing a viable therapeutic target in individuals with metastatic CRC.

## Data Availability Statement

The original contributions presented in the study are included in the article/supplementary material. Further inquiries can be directed to the corresponding authors.

## Ethics Statement

The studies involving human participants were reviewed and approved by The Institutional Ethics Committee of Jiangjin District Central Hospital. The patients/participants provided their written informed consent to participate in this study. The animal study was reviewed and approved by Laboratory Animal Welfare and Ethics Committee of Chongqing University. Written informed consent was obtained from the owners for the participation of their animals in this study.

## Author Contributions

GS and DX conceived and designed the study. JW and FW collected clinical data and analyzed it. SL performed experiments, analyzed data and wrote this manuscript. All authors contributed to the article and approved the submitted version.

## Funding

This study was supported by grants from The General project of Chongqing Natural Science Foundation (Cstc2019jcyj-msxmX0627, Cstc2020jcyj-msxmX0183) and The Talents project of Chongqing, China (CQYC202103063).

## Conflict of Interest

The authors declare that the research was conducted in the absence of any commercial or financial relationships that could be construed as a potential conflict of interest.

## Publisher’s Note

All claims expressed in this article are solely those of the authors and do not necessarily represent those of their affiliated organizations, or those of the publisher, the editors and the reviewers. Any product that may be evaluated in this article, or claim that may be made by its manufacturer, is not guaranteed or endorsed by the publisher.

## References

[1] Bray F Ferlay J Soerjomataram I Siegel RL Torre LA Jemal A. Global Cancer Statistics 2018: GLOBOCAN Estimates of Incidence and Mortality Worldwide for 36 Cancers in 185 Countries. CA Cancer J Clin (2018) 68(6):394–424. doi: 10.3322/caac.21492 30207593

[2] Oki E Ando K Nakanishi R Sugiyama M Nakashima Y Kubo N. Recent Advances in Treatment for Colorectal Liver Metastasis. Ann Gastroenterol Surg (2018) 2(3):167–75. doi: 10.1002/ags3.12071 PMC5980283 29863162

[3] Mongiat M Fu J Oldershaw R Greenhalgh R Gown AM Iozzo RV. Perlecan Protein Core Interacts With Extracellular Matrix Protein 1 (ECM1), a Glycoprotein Involved in Bone Formation and Angiogenesis. J Biol Chem (2003) 278(19):17491–9. doi: 10.1074/jbc.M210529200 12604605

[4] Gao D Ma X Lian P Zhou S Chen J. Pathogenetic Mechanism of Lipoid Proteinosis Caused by Mutation of the Extracellular Matrix Protein 1 Gene. Mol Med Rep (2018) 17(6):8087–90. doi: 10.3892/mmr.2018.8928 29693130

[5] Wang Z Zhou Q Li A Huang W Cai Z Chen W. Extracellular Matrix Protein 1 (ECM1) is Associated With Carcinogenesis Potential of Human Bladder Cancer. Onco Targets Ther (2019) 12:1423–32. doi: 10.2147/OTT.S191321 PMC6389008 30863109

[6] Dai Z Cai L Chen Y Wang S Zhang Q Wang C. Brusatol Inhibits Proliferation and Invasion of Glioblastoma by Down-Regulating the Expression of ECM1. Front Pharmacol (2021) 12:775680. doi: 10.3389/fphar.2021.775680 34970146 PMC8713816

[7] Jeong S Lee SG Kim H Lee G Park S Kim IK. Simultaneous Expression of Long Non-Coding RNA FAL1 and Extracellular Matrix Protein 1 Defines Tumour Behaviour in Young Patients With Papillary Thyroid Cancer. Cancers (Basel) (2021) 13(13):3223. doi: 10.3390/cancers13133223 34203279 PMC8268647

[8] Xiong GP Zhang JX Gu SP Wu YB Liu JF. Overexpression of ECM1 Contributes to Migration and Invasion in Cholangiocarcinoma Cell. Neoplasma (2012) 59(4):409–15. doi: 10.4149/neo_2012_053 22489696

[9] Wang L Yu J Ni J Xu XM Wang J Ning H. Extracellular Matrix Protein 1 (ECM1) is Over-Expressed in Malignant Epithelial Tumors. Cancer Lett (2003) 200(1):57–67. doi: 10.1016/s0304-3835(03)00350-1 14550953

[10] Huang W Huang Y Gu J Zhang J Yang J Liu S. miR-23a-5p Inhibits Cell Proliferation and Invasion in Pancreatic Ductal Adenocarcinoma by Suppressing ECM1 Expression. Am J Transl Res (2019) 11(5):2983–94. PMC6556669 31217868

[11] Lal G Hashimi S Smith BJ Lynch CF Zhang L Robinson RA. Extracellular Matrix 1 (ECM1) Expression is a Novel Prognostic Marker for Poor Long-Term Survival in Breast Cancer: A Hospital-Based Cohort Study in Iowa. Ann Surg Oncol (2009) 16(8):2280–7. doi: 10.1245/s10434-009-0533-2 19521735

[12] Liu LQ Hu L Hu XB Xu J Wu AM Chen H. MiR-92a Antagonized the Facilitation Effect of Extracellular Matrix Protein 1 in GC Metastasis Through Targeting its 3'UTR Region. Food Chem Toxicol (2019) 133:110779. doi: 10.1016/j.fct.2019.110779 31472228

[13] Chen H Jia W Li J. ECM1 Promotes Migration and Invasion of Hepatocellular Carcinoma by Inducing Epithelial-Mesenchymal Transition. World J Surg Oncol (2016) 14(1):195. doi: 10.1186/s12957-016-0952-z 27460906 PMC4962417

[14] Hou YQ Lou JT Peng L Zhou L Ni J Kong XT. Detection and Clinical Significance of ECM1 Gene Expression in Colorectal Cancer Tissue. World Chin J Digestol (2007) 17):1960–4. doi: 10.3969/j.issn.1009-3079.2007.17.015

[15] Hou YQ Zhong RQ Peng L Kong XT. Expression and Diagnosis Value of ECM1 in Colorectal Cancer Tissues. Shandong Med J (2009) 49(52):1–2. doi: 10.3969/j.issn.1002-266X.2009.52.001

[16] Engstrand J Nilsson H Strömberg C Jonas E Freedman J. Colorectal Cancer Liver Metastases - A Population-Based Study on Incidence, Management and Survival. BMC Cancer (2018) 18(1):78. doi: 10.1186/s12885-017-3925-x 29334918 PMC5769309

[17] Dekker E Tanis PJ Vleugels JLA Kasi PM Wallace MB. Colorectal Cancer. Lancet (2019) 394(10207):1467–80. doi: 10.1016/S0140-6736(19)32319-0 31631858

[18] Mathieu E Meheus L Raymackers J Merregaert J. Characterization of the Osteogenic Stromal Cell Line MN7: Identification of Secreted MN7 Proteins Using Two-Dimensional Polyacrylamide Gel Electrophoresis, Western Blotting, and Microsequencing. J Bone Miner Res (1994) 9(6):903–13. doi: 10.1002/jbmr.5650090616 8079665

[19] Yuan S Norgard RJ Stanger BZ. Cellular Plasticity in Cancer. Cancer Discov (2019) 9(7):837–51. doi: 10.1158/2159-8290.CD-19-0015 PMC6606363 30992279

[20] Puisieux A Brabletz T Caramel J. Oncogenic Roles of EMT-Inducing Transcription Factors. Nat Cell Biol (2014) 16(6):488–94. doi: 10.1038/ncb2976 24875735

[21] Dong B Wu Y. Epigenetic Regulation and Post-Translational Modifications of SNAI1 in Cancer Metastasis. Int J Mol Sci (2021) 22(20):11062. doi: 10.3390/ijms222011062 34681726 PMC8538584

[22] Dongre A Weinberg RA. New Insights Into the Mechanisms of Epithelial-Mesenchymal Transition and Implications for Cancer. Nat Rev Mol Cell Biol (2019) 20(2):69–84. doi: 10.1038/s41580-018-0080-4 30459476

[23] Gan L Meng J Xu M Liu M Qi Y Tan C. Extracellular Matrix Protein 1 Promotes Cell Metastasis and Glucose Metabolism by Inducing Integrinβ4/FAK/SOX2/HIF-1α Signaling Pathway in Gastric Cancer. Oncogene (2018) 37(6):744–55. doi: 10.1038/onc.2017.363 29059156

[24] Lee KM Nam K Oh S Lim J Kim RK Shim D. ECM1 Regulates Tumor Metastasis and CSC-Like Property Through Stabilization of β-Catenin. Oncogene (2015) 34(50):6055–65. doi: 10.1038/onc.2015.54 25746001

[25] Song M Bode AM Dong Z Lee MH. AKT as a Therapeutic Target for Cancer. Cancer Res (2019) 79(6):1019–31. doi: 10.1158/0008-5472.CAN-18-2738 30808672

[26] He Y Sun MM Zhang GG Yang J Chen KS Xu WW. Targeting PI3K/Akt Signal Transduction for Cancer Therapy. Sig Transduct Target Ther (2021) 6(1):425. doi: 10.1038/s41392-021-00828-5 PMC8677728 34916492

[27] Noorolyai S Shajari N Baghbani E Sadreddini S Baradaran B. The Relation Between PI3K/AKT Signalling Pathway and Cancer. Gene (2019) 698:120–8. doi: 10.1016/j.gene.2019.02.076 30849534

[28] Jin J Zhang Z Zhang S Chen X Chen Z Hu P. Fatty Acid Binding Protein 4 Promotes Epithelial-Mesenchymal Transition in Cervical Squamous Cell Carcinoma Through AKT/Gsk3β/Snail Signaling Pathway. Mol Cell Endocrinol (2018) 461:155–64. doi: 10.1016/j.mce.2017.09.005 28893569

[29] Zhao GX Xu YY Weng SQ Zhang S Chen Y Shen XZ. CAPS1 Promotes Colorectal Cancer Metastasis *via* Snail Mediated Epithelial Mesenchymal Transformation. Oncogene (2019) 38(23):4574–89. doi: 10.1038/s41388-019-0740-7 30742066

[30] Wang N Song Q Yu H Bao G. Overexpression of FBXO17 Promotes the Proliferation, Migration and Invasion of Glioma Cells Through the Akt/GSK-3β/Snail Pathway. Cell Transplant (2021) 30:9636897211007395. doi: 10.1177/09636897211007395 33853342 PMC8058804

